# Abandonment of Treatment for Latent Tuberculosis Infection and Socioeconomic Factors in Children and Adolescents: Rio De Janeiro, Brazil

**DOI:** 10.1371/journal.pone.0154843

**Published:** 2016-05-05

**Authors:** Angela Marcia Cabral Mendonça, Afrânio Lineu Kritski, Marcelo Gerardin Poirot Land, Clemax Couto Sant’Anna

**Affiliations:** 1 Federal University of Rio de Janeiro, Rio de Janeiro, RJ, Brazil; 2 Academic Tuberculosis Programme, School of Medicine, Federal University of Rio de Janeiro, Rio de Janeiro, RJ, Brazil; 3 School of Medicine, Federal University of Rio de Janeiro, Rio de Janeiro, RJ, Brazil; Johns Hopkins Bloomberg School of Public Health, UNITED STATES

## Abstract

**Background:**

Routine data on the use of isoniazid preventive therapy (IPT) in children and adolescents are scarce in high tuberculosis (TB) burden countries.

**Objective:**

To describe the factors related to abandonment of IPT in children and adolescents with latent tuberculosis infection (LTBI) receiving routine care.

**Methods:**

Retrospective (2005–2009) descriptive study of 286 LTBI cases with indication of IPT and serviced at a pediatric hospital in the State of Rio de Janeiro, Brazil. Survival analysis of the risk of abandonment of IPT over six months was performed, including multivariate analysis using the Cox proportional hazards model.

**Results:**

Out of the 245 cases of LTBI included, 62 abandoned IPT (25.3%; 95% CI: 20%-31%). On multivariate analysis, the variables related to the IPT abandonment hazard ratio were the Human Development Index (HDI) (hazard ratio—HR: 0.004; 0.000–0.569) of the place of residence and the contact with adults that were not undergoing anti-TB treatment (HR: 7.30; 1.00–53.3).

**Conclusion:**

This study reveals the relevance of the relation of abandonment of IPT to the socioeconomic conditions at the place of residence and poor adherence to the active TB treatment. Educational measures to stimulate preventive treatment of child contacts and curative treatment of index cases should target the full familial setting.

## Introduction

Tuberculosis (TB), caused by *Mycobacterium tuberculosis* (*Mtb*), still poses a major health problem worldwide [1]. According to the World Health Organization (WHO) estimates, nine million cases of TB occurred in 2013, mostly in low- and middle-income countries [1]; of these cases, 550,000 patients were children, and 80,000 of them died from TB [1, 2].

WHO considers identification and treatment of latent TB infection (LTBI) a tool for global TB control, especially in high-risk groups, such as children under five years old, individuals infected with the human immunodeficiency virus (HIV), and patients undergoing immunosuppressive therapy [1, 3].

Approximately 5 to 10% of LTBI cases might progress to active TB at some point in life, while that proportion might be as high as 40% among children under five years old [3]. Treatment of LTBI with isoniazid in the childhood reduces the odds of progression to active TB to less than 0.5%. In Brazil, the isoniazid preventive therapy (IPT) lasts six months and is available at no cost [4, 5].

The literature includes a small amount of data on IPT abandonment, tolerance and effectiveness under routine care [5–10]. The global rates of IPT abandonment range from 20 to 80%, with the lowest proportions corresponding to clinical research sites [11]. In Uganda, Indonesia and Africa, the rates of IPT abandonment by child contacts of pulmonary TB patients under routine care were 46%, 74% and 80%, respectively [12–14].

WHO promotes health actions targeting the social determinants of TB to achieve full control of this condition [1]. The Human Development Index (HDI), Gini index and income *per capita* have been used for evaluating social determinants. HDI is the most widespread measure that captures the socioeconomic inequality and human development used specially by United Nations. One of its advantages is the multidimensionality, using information based on income, education and health within the complex context of large urban centers [15–17]. Some studies found that the incidence of TB decreases faster in countries with high HDI, low child mortality and wide access to healthcare services [, 16,17].

The BRICS (Brazil, Russia, India, China and South Africa) emerging economies have undergone deep economic, social, political and health-related changes in the past years [15]. Those changes affected HDI, which allows for estimating the access of a given population to health, education and income [16]. Brazil and China are among the 22 countries in which the number of TB cases exhibited steady declines over the past 20 years [1, 15], which might be associated with economic growth and a reduction of social inequalities.

In the last 15 years, a conditional cash transfer program was incorporated in the social security system and now it benefits around a quarter of Brazilian population. In addition to this income distribution process, Brazil increased the health system access through nationwide primary care strategies, including the Health Family Program [15,16]

The aim of this present study was describing the factors related to IPT abandonment among children and adolescents residing in the State of Rio de Janeiro, Brazil, which displayed the second highest incidence of TB in Brazil in 2012 (65.6/100,000 inhabitants) [18]. In 2010, the city of Rio de Janeiro’s HDI was 0.761 and, thus, better if compared to 2004 (0.664) [19]. The state of Rio de Janeiro’s HDI was 0.811 in 2000 and 0.852 in 2007, exhibiting thus gradual improvement [19].

## Methods

This present retrospective, longitudinal and descriptive study was conducted at the Jesus Municipal Hospital (HMJ), Rio de Janeiro, Brazil. HMJ is an exclusively pediatric healthcare facility and is a reference hospital in the state of Rio de Janeiro for the investigation and treatment of all pediatric diseases, including TB associated or not with HIV infection. The hospital performs almost 90,000 outpatients’ visits and 5,000 admissions per year.

Individuals with indication of IPT for LTBI at HMJ from January 2002 to December 2009 were considered as eligible for this study. Inclusion criteria: individuals under 15 years old, residing in the city of Rio de Janeiro. Exclusion criteria: the ones who did not return for the first monthly follow-up visit after the IPT indication (non-adherence) and the cases that progressed to active TB while undergoing IPT.

The definition by Solomon at al. [20] distinguishing non-adherence from lack of persistence (abandonment) is assumed. According the authors, there are two types of non-adherence, i.e., primary, “when patients do not even fill a new prescription” and secondary, “when prescriptions are filled, but the medication is not taken as prescribed”. In contrast, the lack of persistence is characterized “when patients, who may have perfect primary and secondary adherence, self-discontinue therapy by not refilling their prescriptions”.

Active TB was ruled out in all eligible subjects based on their clinical and epidemiological history, physical exam and chest x-rays, as well as gastric lavage and/or sputum induction for acid-fast bacteria visualization and culture whenever necessary and possible.

The participants’ socio-demographic and clinical-epidemiological data were collected and 10% of body weight cuts percentile was used according to routine hospital data collection to differentiate eutrophic from malnourished children. The type of IPT outcome was assessed in the full sample, as well as the proportion of TB cases after IPT (isoniazid [INH] over six months) completion [use of INH for 6 months] or abandonment. IPT schedule was based on a monthly self-administered INH. Follow-up was performed for at least two years through the Notifiable Diseases Information System (SINAN)–the Brazilian system for registering and processing data on notifiable diseases—or visits to HMJ after IPT abandonment. In the cases featuring history of contact with adults having pulmonary TB, it was identified whether the index case was or was not under anti-TB treatment.

At the time of the study, IPT was indicated according to the Brazilian Health Ministry (MoH-Brazil) guidelines from 2002 to 2009 [21]. The tuberculin skin test (TST) was performed using purified protein derivative (PPD) Rt-23 (*State Serum Institute*, Copenhagen, Denmark).

The indications of IPT in the children's age group at the time of the study were: children and adolescents under 15 years without signs consistent with active TB,

1. With prior contact with pulmonary tuberculosis
  - Not vaccinated with BCG or vaccinated more than two years ago and TST ≥ 10 mm
  - Vaccinated with BCG less than two years ago and TST ≥ 15 mm;
2. With recent TST conversion (up to 12 months);
3. Immunosuppressed with pulmonary tuberculosis household contact;
4. TST≥ 10 mm with clinical conditions such as corticosteroids users in immunosuppressive doses or other immunosuppressive drugs.

Possible adverse effects of IPT (abdominal pain, vomiting and hepatitis) were fetched and registered.

The distance from the participants’ homes to HMJ by car, in kilometers, was calculated by means of *Google Maps* or a Global Positioning System (GPS). Those softwares were also used for estimating the number of bus rides required to commute between the participants’ homes and HMJ. HDI at the participants’ place of residence was assessed based on the 2000 United Nations Development Programme (UNDP) [19].

The database was created by using Access software (Microsoft, Redmond, WA, USA), and data analysis was performed using the Statistical Package for the Social Sciences version 22.0 for Windows (SPSS Inc., Chicago, IL, USA).

In the statistical analysis, the hazard ratios of several socio-demographic and clinical-epidemiological variables relative to IPT abandonment were used in survival analysis and in the Cox proportional hazards semi-parametric model. The IPT abandonment-free survival was considered for the analysis, i.e., the situation in which after having started IPT, participants did not return to the healthcare unit for more than 30 consecutive days after the follow-up visit scheduled. The IPT abandonment time variable, expressed in months, was calculated from the date of IPT onset to the IPT abandonment date.

Cox models were used to calculate the univariate hazard ratio of the variables related to risk of IPT abandonment. The variables with 80% significance (p-value < 0.20) were selected for multivariate analysis. After the assessment of the multicollinearity among every possible candidate variables, a parsimonious model was built through the backward elimination method, using the likelihood ratio test as selection criteria (for further analysis of the method see Kleimbaum et al[22] and Greenland [23]). A more parsimonious model was selected by means of the likelihood ratio test. The curves of cumulative risk and median time to IPT abandonment were estimated by means of the Kaplan-Meier (KM) method.

The database is in S1 Dataset.

### Ethics Statement

The study was approved by the ethical committee of the Clementino Fraga Filho University Hospital, School of Medicine, Federal University of Rio de Janeiro, research protocol no. 068/11 CAAE. Because of the retrospective nature of the study and the disconnection from personal information, the requirement to obtain informed consent from the legal persons responsible for the individuals under study was waived by our institutional review board. It is a merely observational and descriptive retrospective analysis, and it does not offer physical and/or biological risk. The confidentiality of personal identification of individuals was assured by the researchers as well. To ensure confidentiality, the data were completely delinked from any personal identifiers before they were analyzed.

## Results

A total of 286 cases of LTBI with indication of IPT was evaluated. Out of such, 41 (14.3%) cases were excluded for the following reasons: 37 (12.9%) subjects did not return for the first monthly follow-up visit; and four (1.4%) progressed to active TB while undergoing IPT. The socio-demographic and clinical-epidemiological characteristics of the 245 individuals included in the study are described in Table 1.

**Table 1:** Distribution of 245 individuals diagnosed with LTBI and with indication of IPT included in the study according to socio-demographic and clinical epidemiological characteristics. HMJ-RJ, 2002–2009.

CHARACTERISTICS	N	%
**Gender**		
**Female**	**121**	**49.4**
**Male**	**124**	**50.6**
**Age**		
**<6 months old**	**7**	**2.9**
**≥6 months-1 year old**	**13**	**5.3**
**≥1-5years old**	**79**	**32.2**
**≥5-10years old**	**115**	**46.9**
**≥10years old**	**31**	**12.7**
**Place of residence**		
**City of Rio de Janeiro**	**199**	**81.2**
**State of Rio de Janeiro**	**46**	**18.8**
**Number of different transport means from the place of residence to HMJ (in the same occasion)**		
**1**	**132**	**53.9**
**≥2**	**113**	**46.1**
**HIV sero status**		
**Positive**	**16**	**23.5**
**Negative**	**52**	**76.5**
**N/A**	**177**	
**BCG vaccination**		
**Yes**	**223**	**99.6**
**No**	**1**	**0.4**
**N/A**		
	**21**	
**Bodyweight (percentile)**		
**≥10**	**230**	**93.9**
**<10**	**15**	**5.1**
**TST result (mm)**		
**>5–10**	**14**	**6.9**
**≥10**	**190**	**93.1**
**IPT adverse effects**		
**Yes**	**8**	**3.3**
**No**	**237**	**96.7**
**Contact with pulmonary TB**		
**Yes**	**227**	**92.7**
**No**	**18**	**7.34**
**Outcome**		
**IPT completed**	**183**	**74.7**
**IPT abandonment**	**62**	**25.3**

HMJ—Jesus Municipal Hospital

N/A—not available

TST—tuberculin skin test

TB—tuberculosis

IPT—isoniazid preventive therapy

Approximately 92.7% (227/245) of the cases with indication of IPT corresponded to individuals with positive TST results and histories of contact with adults with pulmonary TB. In the cases remaining, IPT was indicated due to the presence of immunosuppressive conditions, regardless of the TST results.

Adverse reactions to IPT were identified in 8 (3.3%) individuals (7 under 10 years old; 1 had HIV co-infection), including 5 cases of abdominal pain and 3 of vomiting after taking INH. The occurrence of adverse effects did not require discontinuation of IPT in any cases.

TST was performed for 96.3% (236/245) of the sample. Indurations were lower than 5 mm in 13.6% of the cases (32/236); a second TST was performed in 29 of those individuals six to eight weeks after IPT initiation at HMJ to detect tuberculin conversion, which occurred in 19 (65.5%) cases.

In 178 instances (78.4%), the index case was undergoing anti-TB treatment. Twenty-four (9.8%) individuals had contact with multidrug-resistant TB (MDR-TB); none of them exhibited progression to active TB during the two-year follow-up.

Most participants resided in the city of Rio de Janeiro (81.2%); the HDI mean of the place of residence was 0.807 ± 0.071 and the HDI median of the place of residence was 0.807 (minimum 0.659- maximum 0.959).

The rate of IPT abandonment was 25.3% (62/245), distributed as follows: 43.5% (27/62) in the second month of treatment, 35.5% (22/62) in the third month, 16.1% (10/62) in the fourth month and 4.8% (3/62) in the fifth month.

Two cases progressed to active TB after IPT abandonment. One individual had pulmonary TB four months after IPT abandonment, and the other developed the peripheral lymph node form of disease 16 months after discontinuation of the treatment.

Among the 183 individuals who completed IPT, 2 (1%) progressed to pulmonary TB 34 and 60 months after the end of treatment, respectively. Both were non HIV-infected children and were exposed to other adult with TB. The diagnosis of active TB was established at HMJ.

**Fig 1:**
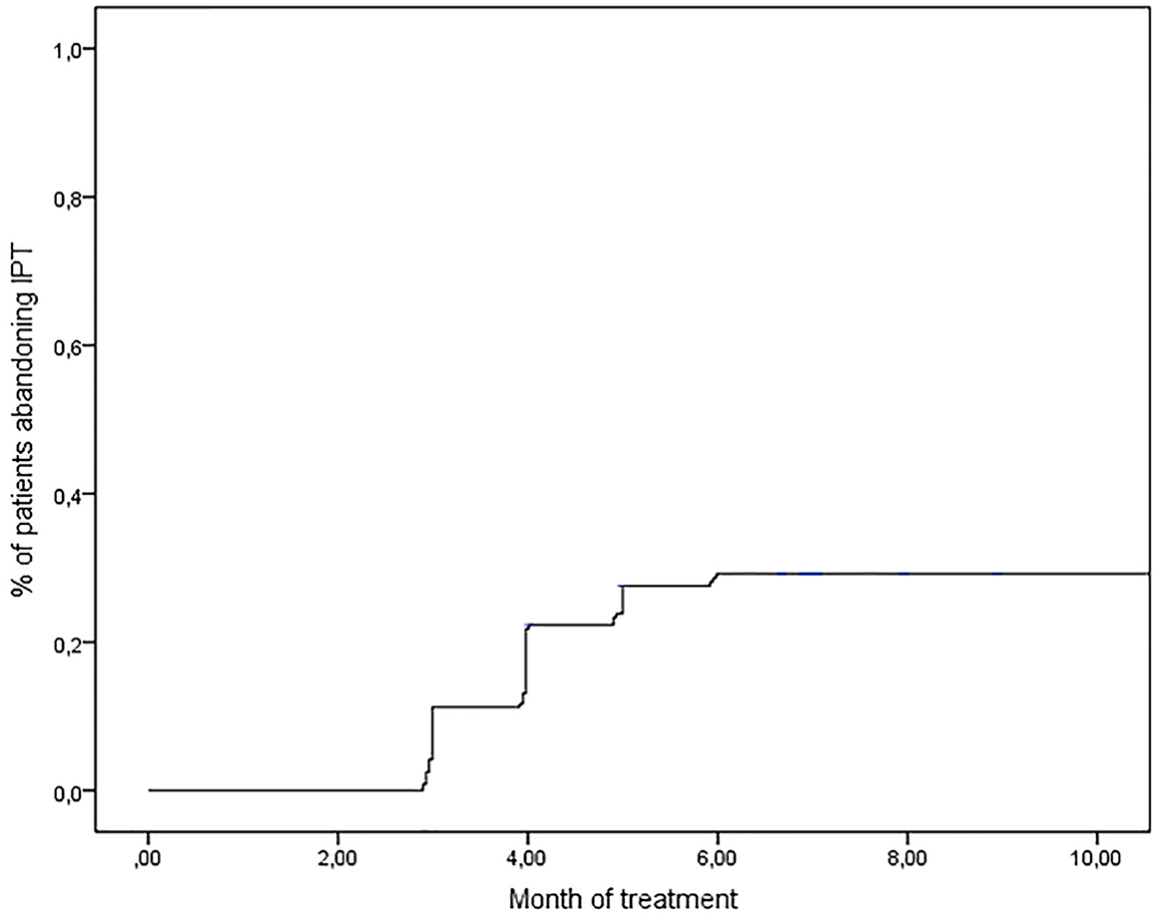
Plot for the cumulative risk of IPT abandonment. HMJ-RJ, 2002–2009.

The cumulative risk for IPT abandonment over six months was of approximately 25.3% (CI: 20%-31%). (Fig 1)

Table 2 describes the analysis of the hazard ratios for IPT abandonment-free survival corresponding to the participants in this study.

**Table 2:** Univariate Cox proportional hazards model for IPT abandonment-free survival of children and adolescents with LTBI in the state of Rio de Janeiro, 2002–2009.

VARIABLE	HR	(95%) CI	P
**Gender (n = 245)**			
**Female/Male**	**0.771**	**0.46–1.27**	**0.30**
**Age range (n = 245)**			
**Children (< 10 years old)/ Adolescents (**≥**10 years old)**	**1.381**	**0.70–2.73**	**0.34**
**Contact with pulmonary TB (n = 245)**			
**Yes/No**	**2.521**	**0.62–10.3**	**0.20**
**Contact with index case that was not undergoing anti-TB treatment (n = 202)**			
**Yes/No**	**7.871**	**1.08–57.3**	**0.04**
**Body weight (percentile) (n = 245)**			
≥**10/<10**	**1.321**	**0.41–4.22**	**0.64**
**Tuberculin skin test (mm) (n = 236)**			
≥**10/<10**	**1.401**	**0.69–2.34**	**0.31**
**IPT adverse effects (n = 245)**			
**Yes/No**	**1.831**	**0.57–5.84**	**0.30**
**HIV serum status (n = 68)**			
**Positive/Negative**	**0.331**	**0.76–1.41**	**0.14**
**Place of residence (n = 286)**			
**City of Rio de Janeiro/Out of the City of Rio de Janeiro**	**1.421**	**0.70–2.89**	**0.32**
**Place of residence’s HDI (n = 245)**	**0.04**	**0.00–0.42**	**0.02**
**Driving distance between place of residence and HMJ (km) (n = 245)**	**1.02**	**0.99–1.04**	**0.06**
**Number of different transport means from the place of residence to HMJ (n = 245)**			
≥**2/<2**	**1.291**	**0.91–1.83**	**0.14**

CI—confidence interval

HDI—human development index

HIV—human immunodeficiency virus

HMJ—Jesus Municipal Hospital

HR—hazard ratio

TB—tuberculosis

IPT—isoniazid preventive therapy

The variables related to the IPT abandonment time at the established significance level (p < 0.20) were contact with an adult that was not undergoing anti-TB treatment (hazard ratio—HR: 7.87; 1.08–57.3); positive HIV serostatus (HR: 0.33; 0.76–1.41); HDI of the place of residence (HR: 0.04; 0.00–0.42); driving distance between place of residence and HMJ (HR: 1.02; 0.99–1.04); and number of different transport means from the place of residence to HMJ (HR: 1.29; 0.91–1.83).

Table 3 describes the results of the multivariate analysis, showing that the following variables remained significantly related to the IPT abandonment time: HDI of the place of residence (HR: 0.004; 0.000–0.569) and contact with an adult that was not undergoing anti-TB treatment (HR: 7.30; 1.00–53.3).

**Table 3:** Multivariate Cox proportional hazards model for IPT abandonment-free survival of children and adolescents with LTBI in the state of Rio de Janeiro, 2002–2009.

VARIABLE	HR	(95%) CI	P
**Place of residence’s HDI**	**0.004**	**0.000–0.569**	**0.029**
**Contact with untreated pulmonary TB**	**7.30**	**1.00–53.3**	**0.050**

CI- confidence interval

HDI- human development index

HMJ- Jesus Municipal Hospital

HR- hazard ratio

TB- tuberculosis

## Discussion

A total of 245 individuals diagnosed with LTBI was analysed; IPT was predominantly indicated for individuals aged 5–10 years old or who had contact with pulmonary TB. In turn, the indication of IPT for children of 5 years and older was based on the individual consideration of each case and corresponding risk factors [6, 22, 23]. At the time this present study was performed, as in the present, the official standards allowed for broader-scoped indication of IPT for contacts of TB from various age ranges to increase the protection coverage [, 24, 25].

The frequency of adverse effects displayed in this study was approximately 3% and was greater in children under 10 years old. The most common adverse reaction among children was the difficulty of ingesting the INH tablet. This problem was less frequent among adolescents. None of them required IPT discontinuation similarly to what was observed by David *et al*. [6] in Brazil and Young *et al*. [26], in Mexican immigrant children/USA. The present study shows the low toxicity of INH in children and adolescents.

In this present case series, more than 90% of the individuals with indication of IPT had a history of contact with pulmonary TB; this proportion is similar to the one reported by Gomes *et al*. [8] in Brazil (97%) but higher than the one reported by Dobler *et al*. [27] in Australia (69%). In the cases without contact with TB, the indication of IPT was due to immunosuppressive diseases, as Dobler *et al*. [27] also reported.

Only 4% of the individuals included in our case series did not perform TST, the reason being the lack of large-scale availability in the first quarter of 2003 (due to administrative reforms at *State Serum Institute*, Copenhagen, Denmark). The high risk of LTBI and active TB exhibited by children exposed to pulmonary TB might justify the indication of IPT made at HMJ for individuals not subjected to TST, according to WHO recommendations [28, 29].

Approximately 10% of the individuals of this present study had contact with MDR- TB index cases. This proportion is smaller than that found in Angola by Fortunato *et al*. [30], where 18% of the index cases of child contacts with indication of IPT exhibited MDR-TB. That discrepancy might be accounted for by the differences in the incidence of TB between Angola and Brazil. In our case series, no contact with an MDR-TB index case progressed to active TB in the two-year follow-up after completing the IPT. This finding disagrees with the results of Sneag *et al*.’s [31] study in the USA, in which all five children exposed to MDR-TB progressed to active TB after IPT.

Different from the Brazilian guidelines at the time when the study was performed, WHO recommended that child contacts of MDR-TB cases should be followed up for two years without IPT [1, 29].

Approximately 25% of the individuals in our case series abandoned IPT under routine care. Thus, our findings agree with results for Guinea-Bissau (24%) [32] and Brazil (32–33%) [5, 8] but are lower than the rates reported in the USA (60%), Africa (46%) and Indonesia (74%) by Li *et al*. [33], Zyl *et al*. [12] and Rutherford *et al*. [14, 34], respectively.

In this present study, contact with adults that were not undergoing anti-TB treatment had significant association with IPT abandonment. In a qualitative study conducted in Indonesia, Rutherford *et al*. [34] observed that the children’s adherence to IPT was related to their caregivers’ own experience with TB (i.e., the desire to spare the children a similar experience and a better understanding of the disease severity). The medical prescription of IPT should consider factors like the families’ comprehension of the disease and their social and economic status.

According to a qualitative study assessing factors related to IPT abandonment in Brazil, the ones most frequently mentioned were lack of money to pay for transportation to healthcare services, drug presentation as tablet (inadequate for children); and absence of disease [8].

HDI corresponding to the participants’ place of residence was significantly related to IPT abandonment (HR: 0.004; 0.000–0.569), suggesting that high HDI is a protective counter factor. These findings agree with other Brazilian study that found inverse relationship between HDI and severity of TB disease [35].

Some influence of HDI on the epidemiological indicators of active TB and LTBI in childhood and adolescence might be assumed. As TB is mainly associated with low income and low educational levels (despite all advances and improvements in the access to treatment), populations with lower socioeconomic status represent high-risk groups for infection, disease, and abandonment of treatment [7].

In our analysis, most individuals with LTBI resided in the city of Rio de Janeiro, particularly, in the neighborhoods with the lowest HDIs; those findings agree with the ones reported by Souza *et al*. [36] in Manaus, Brazil, where the highest concentration of smear positive TB cases were found in the neighborhoods with deficient urbanization and lowest HDI. The incidence of TB in state of Rio de Janeiro was the highest in Brazil throughout the study period, albeit with a gradual annual reduction [18]. As far as the indicators of living conditions regards, the HDI varied from 0.811 in 2000 to 0.852 in 2007 [19]. One might assume a strong relationship between improvement of HDI and incidence of TB in the city of Rio de Janeiro.

The discrepancy among the rates of treatment abandonment described in the literature [5, 8, 12, 14, 32–34] suggests that IPT abandonment is not exclusively associated with socioeconomic factors. In addition, ensuring proper diagnosis and treatment does not suffice to control TB; one must still overcome the psychological challenge posed by having to convince people (or caregivers, in the case of children) of the need to treat a non-contagious infection in the absence of symptoms of disease [5, 29].

Silva *et al*. [7] in Rio de Janeiro, Brazil, performed a qualitative analysis of the reasons adduced by caregivers not to administer IPT to children and found that the following barriers to treatment included need to work and being unable to administer IPT: (1) lack of time to schedule medical visits for the children and to pick up the medication due to job-related factors and (2) absence of symptoms.

In this present study, progression to active TB occurred in 2% of the cases that abandoned IPT and in 1% of the individuals with full adherence after the end of treatment. The individuals who finished IPT were exposed to another TB source. It is compatible with a TB exogenous new infection. We were not able to identify any Brazilian study assessing progression to active TB among children and adolescents undergoing IPT. Marais *et al*. [13] reported that 3% of the children who abandoned IPT in South Africa exhibited progression to active TB. According to Fortunato *et al*. [30], in Angola, 32% of LTBI cases progressed to active TB after the end of IPT. Those authors provided no explanation for such a high proportion of cases progressing to active TB after the end of IPT but speculated on the possibilities of new exposure to *Mtb* or to MDR-TB.

A study performed over a 30-year period in USA with 1,882 children with LTBI subject to IPT found that only 8 (0.4%) progressed to active TB after the end of IPT [11]. Those cases were explained as likely cases of new infection, perhaps the same reason found in this study.

WHO promotes social protection as one of the three pillars for global control of TB and treatment of LTBI in vulnerable groups [2, 29, 37]. High levels of social protection might increase access to healthcare services, thus facilitating the diagnosis of cases, and providing stable social conditions favorable to the completion of treatment [38, 39]. One study performed in the European Union found that social protection had an inverse correlation with TB incidence and mortality [37].

One of the limitations of this present study is that the population under analysis consisted of children and adolescents serviced at HMJ only. Therefore, it is not possible to make inferences for the full universe of individuals with LTBI from the same age group and residing in the city of Rio de Janeiro. In addition, this present study has the same limitations as any study based on secondary data resulting from incomplete information. As well, it should be noted that HDI is an ecologic measure, and further prospective studies should correlate this measure to other more individual.

This study emphasizes the fact that public policies might play a relevant role in the prevention and control of TB in areas with high disease burden. Investment in social protection is fully justified not only because it is fair to the vulnerable population but also because it contributes to nationwide TB control. This study was able to demonstrate how these inequalities may have determined behaviors that hinder the implementation of prevention strategies for endemic diseases such as TB. One can foresee that the socio-economic improvements and reduction of local inequalities can have great impact on regional public policy in Brazil.

## Supporting Information

S1 Dataset Database for publication.sav. (SAV)
